# A Step-by-Step Refined Strategy for Highly Efficient Generation of Neural Progenitors and Motor Neurons from Human Pluripotent Stem Cells

**DOI:** 10.3390/cells10113087

**Published:** 2021-11-09

**Authors:** Jie Ren, Chaoyi Li, Mengfei Zhang, Huakun Wang, Yali Xie, Yu Tang

**Affiliations:** 1 Department of Geriatrics, Xiangya Hospital, Central South University, Changsha 410008, China; 198111024@csu.edu.cn (J.R.); lcylnsn@csu.edu.cn (C.L.); 208111028@csu.edu.cn (M.Z.); whk4311579@outlook.com (H.W.); 2 Aging Research Center, Xiangya Hospital, Central South University, Changsha 410008, China; 3 National Clinical Research Center for Geriatric Disorders, Xiangya Hospital, Central South University, Changsha 410008, China; xieyali@csu.edu.cn; 4 The Biobank of Xiangya Hospital, Central South University, Changsha 410008, China; 5 Department of Neurology, Xiangya Hospital, Central South University, Changsha 410008, China

**Keywords:** pluripotent stem cells, neural progenitor cells, neural differentiation, RA, SMAD, motor neuron

## Abstract

Limited access to human neurons, especially motor neurons (MNs), was a major challenge for studying neurobiology and neurological diseases. Human pluripotent stem cells (hPSCs) could be induced as neural progenitor cells (NPCs) and further multiple neural subtypes, which provide excellent cellular sources for studying neural development, cell therapy, disease modeling and drug screening. It is thus important to establish robust and highly efficient methods of neural differentiation. Enormous efforts have been dedicated to dissecting key signalings during neural commitment and accordingly establishing reliable differentiation protocols. In this study, we refined a step-by-step strategy for rapid differentiation of hPSCs towards NPCs within merely 18 days, combining the adherent and neurosphere-floating methods, as well as highly efficient generation (~90%) of MNs from NPCs by introducing refined sets of transcription factors for around 21 days. This strategy made use of, and compared, retinoic acid (RA) induction and dual-SMAD pathway inhibition, respectively, for neural induction. Both methods could give rise to highly efficient and complete generation of preservable NPCs, but with different regional identities. Given that the generated NPCs can be differentiated into the majority of excitatory and inhibitory neurons, but hardly MNs, we thus further differentiate NPCs towards MNs by overexpressing refined sets of transcription factors, especially by adding human *SOX11*, whilst improving a series of differentiation conditions to yield mature MNs for good modeling of motor neuron diseases. We thus refined a detailed step-by-step strategy for inducing hPSCs towards long-term preservable NPCs, and further specified MNs based on the NPC platform.

## 1. Introduction

One decade ago, the emerging technology of human-induced pluripotent stem cells (iPSCs) reprogrammed directly from adult somatic cells such as primary fibroblasts opened a new era of stem cell biology, which has been rapidly applied in both basic science and translational medicine with the concerted efforts of biologists, clinic doctors and entrepreneurs. Human pluripotent stem cells (hPSCs), including embryonic stem cells (ESCs) and generated iPSCs from patients, either with or without genetic variants, can be further induced into neural progenitors and multiple neuron subtypes, in the manner of the principles of embryonic development [1]. The differentiated neural progenitors and neurons are versatile for studying the molecular biology of neural induction [2,3,4] and transplantation-based cell therapy [5,6], as well as modeling the pathogenesis of diseases [7,8,9,10] and drug screening [11,12]. Those endeavors are especially useful in studying neurodegenerative diseases since murine models might not always truly recapitulate the human pathogenesis, due to species differences, and also previous efforts have been hindered by limited access to patient neurons. Therefore, it is pivotal to establish robust and highly efficient methods of neural differentiation from hPSCs, especially patient-derived iPSCs.

One classical method was inducing hPSCs into floating embryoid bodies (EBs) and further primitive neural tube-like structures or neural rosettes, which is inefficient and labor-intensive for neural induction [12,13,14,15,16]. The second method is that hPSCs are cocultured with particular stromal cell lines such as PA6 and MS5 to initiate neural differentiation, which may introduce uncertain stromal factors of non-human origin [17,18,19,20]. Along with the dissection of triggering signal pathways for neural commitment, scientists in the last decade were dedicated to inducing neural differentiation in an adherent monolayer culture system by key instructive factors and small molecules such as retinoic acid (RA), TGF-β/SMAD inhibitors, bone morphogenic protein (BMP)/SMAD inhibitors, purmorphamine (Pur)/sonic hedgehog (SHH), GSK3β inhibitors like CHIR99021 and fibroblast growth factor 8 (FGF8), among others. In this regard, it is imperative to treat hPSCs with the addition of instructive factors, or the removal of maintenance factors governing pluripotency and other germ layers. RA, as one of the classical instructive factors, was recognized as a neural and pan-neuronal differentiation signal [21], and could be combined with other small molecules to direct hPSCs to the neural lineage [13,14,15,22]. Moreover, the inhibition of dual SMAD signaling (TGF-β/SMAD and BMP/SMAD) was employed to inhibit the pluripotency of hPSCs and further induce rapid and nearly complete neural conversion [23,24,25,26].

In our study, we refined a protocol for rapid differentiation of hPSCs towards neural progenitor cells (NPCs) within 18 days, combining the adherent and neurosphere-floating methods. This protocol makes use of RA signaling and dual-SMAD inhibition (SMADi), respectively, for the adherent treatment, and further induces neurospheres for suspension culture. Both methods could give rise to highly efficient and complete generation of preservable NPCs, without using feeder cells and manual rosette picking. We further compared the different identities of generated NPCs under those two conditions, which might be used for different purposes. It is a robust and versatile protocol for neural induction of hPSCs, either derived from normal or diseased patients. Moreover, it is compatible with other chemical inhibitions for further rostro-caudal patterning and dorso-ventral patterning, so as to obtain different neuron subtypes.

However, since the generated NPCs by both methods are basically with forebrain identities, they can be differentiated into the majority of excitatory and inhibitory neurons, but hardly motor neurons (MNs). In all regards, it has long been tricky for MN differentiation. Although multiple methods have been developed for MNs, including the methods through EBs/MN patterning [27,28], the overall efficiencies are rather low. During recent years, by introducing transcription factors directly into hPSCs, we are able to rapidly differentiate neurons [29,30]. However, it is a one-time process without the intermediate stage that requires repeated differentiation directly from hPSCs. We thus, based on the established preservable NPC platform, further differentiated MNs by introducing transcription factors that govern the development of MNs. We refined sets of transcription factors, especially by adding human *SOX11*, based on our and others’ previous studies [9,31]. We further improved a series of differentiation conditions to yield mature MNs with high efficiencies (~90%), which were later well-used for modeling motor neuron diseases (MNDs), such as amyotrophic lateral sclerosis (ALS).

Overall, we refined a detailed step-by-step protocol for inducing hPSCs towards NPCs, further specified as MNs. The overall strategy will rapidly and efficiently produce ample long-term preservable NPCs for neuron differentiation, and also provide a well-starting NPC pool for differentiating MNs and other neural subtypes. Moreover, based on our refined culture conditions, other neural subtypes are likely to be induced with high efficiencies that are comparable with those in differentiating MNs. We detailed this step-by-step protocol so that researchers, including novices, can also repeat the process from scratch.

## 2. Materials and Methods

### 2.1. General Supplies

1. Cell culture incubator (ThermoFisher, Waltham, MA, USA, HERAcell 150i).
2. 6-well cell culture plates (Jet Biofil, Guangzhou, China, #TCP010006).
3. 24-well cell culture plates (Jet Biofil, #TCP010024).
4. Cryotube vials (ThermoFisher, #375418PK).
5. 15 mL conical tubes (Jet Biofil, #CFT-011150).
6. Coverslips for cell culture (Citotest, Haimen, China, #0346-0910).
7. Microscope slides (Citotest, #188105W).
8. DPBS (Beyotime, Shanghai, China, #C0221D).
9. 96-well Q-PCR plates (Axygen, Union City, CA, USA, #PCR-96M2-HS-C).
10. ABI QuantStudio Dx (Applied Biosystems, Waltham, MA, USA).
11. Fluorescence microscope (Leica, Wetzlar, Germany, DMi8).
12. MultiClamp 700B amplifier (Molecular Devices, San Jose, CA, USA).
13. Clampex10.3 software (Molecular Devices).

### 2.2. Cell Lines Used

1. iPSC-WT (Nuwacell, Hefei, China, #RC01001-A; #RC01001-B).
2. iPSC-ALS (Coriell, Camden, NJ, USA, #ND35660; #ND35664).
3. iPSC-DYT1 (CSUi002-A) [32].
4. iPSC-HD (Coriell, #GM23225).

### 2.3. Culture and Maintenance of hPSCs

1. hPSC medium: ncEpic hPSC Medium (Nuwacell, #RP01001).
2. hPSC Dissociation Buffer (Nuwacell, #RP01007).
3. Accutase (Millipore sigma, Burlington, MA, USA, #SCR005).
4. Matrigel (Corning, Kakegawa, Japan, #354277); aliquot and store at −80 °C.
5. Y-27632 (Selleck, Houston, TX, USA, #S1049); aliquot and store at −80 °C.

### 2.4. Differentiation of hPSCs to NPCs

1. SB431542 (Sigma, St. Louis, MO, USA, #S4317); aliquot and store at −80 °C; avoid exposure to light.
2. LDN193189 (Miltenyi, Bergisch Gladbach, Germany, #130-103-925); aliquot and store at −80 °C; avoid exposure to light.
3. MGCD0103 (Selleck, #S1122); aliquot and store at −80 °C.
4. RA (Sigma, #R2625); aliquot and store at −80 °C; avoid exposure to light.
5. Knockout serum replacement (KOSR, ThermoFisher, #A3181502); aliquot and store at −80 °C.
6. DMEM/F12 (Procell, Wuhan, China, #PM150312).
7. Neurobasal medium (ThermoFisher, #21103049).
8. GlutaMax (Gibco, Amarillo, TX, USA, #35050061).
9. NEAA (Gibco, #11140050).
10. β-ME (Procell, #PB180633).
11. Penicillin/Streptomycin (P/S, Solarbio, Beijing, China, #P1400).
12. DMSO (Solarbio, #D8371).

### 2.5. Differentiation of NPCs to Neurons

1. DMEM (Procell, #PM150210).
2. DMEM/F12 (Procell, #PM150312).
3. Neurobasal medium (ThermoFisher, #21103049).
4. Fetal bovine serum (FBS, TransGen Biotech, Beijing, China, #PS201-02); aliquot and store at −80 °C.
5. Poly-L-ornithine (PLO, Sigma, #P4957).
6. Mouse laminin (Corning, #354232); aliquot and store at −80 °C.
7. Fibronectin (R&D Systems, Tokyo, Japan, #1918-FN); aliquot and store at −80 °C.
8. AraC (Sigma, #C1768); aliquot and store at −80 °C.
9. Purmorphamine (Calbiochem, San Diego, CA, USA, #540220); aliquot and store at −80 °C.
10. B27 supplement (Gibco, #17504044).
11. N2 supplement (Gibco, #17502048).
12. L-ascorbic acid (L-AA, Sigma, #A4403); aliquot and store at −80 °C.
13. Forskolin (Sigma, #F6886); aliquot and store at −80 °C.
14. Puromycin (Solarbio, #P8230); aliquot and store at −80 °C.
15. BDNF (Novoprotein, Shanghai, China, #C076); aliquot and store at −80 °C.
16. GDNF (Novoprotein, #C226); aliquot and store at −80 °C.

### 2.6. Immunostaining

1. 4% Paraformaldehyde (PFA, Servicebio, Wuhan, China, #G1101).
2. Triton X-100 (Solarbio, #T8200).
3. Tween-20 (Macklin, Rochelle, IL, USA, #C10232628).
4. Bovine serum albumin (BSA, BioFroxx, Guangzhou, China, #4240).
5. Hoechst 33342 (Beyotime, Shanghai, China, #C1025).
6. Poly(vinyl alcohol) (PVA, Macklin, P816862).
7. Primary and secondary antibodies (Table A1).

### 2.7. Q-PCR

1. TransZol (TransGen Biotech, #ET101-01).
2. RevertAid First Strand cDNA Synthesis Kit (ThermoFisher, #K1622).
3. PerfectStart Green qPCR SuperMix (TransGen Biotech, #AQ601-01).
4. Primers (Table A2).

### 2.8. Medium and Solution Setup

1. KOSR Medium: DMEM/F12 medium with 20% KOSR, 1% GlutaMax, 1% NEAA, 50 µM β-ME and 1% P/S.
2. NSP Medium: DMEM/F12 medium containing 1% N2, 1% GlutaMax, 1% NEAA, 50 µM β-ME, 1% P/S, 8 µg/mL Heparin, 20 ng/mL bFGF and 20 ng/mL EGF.
3. NPC Medium: DMEM/F12 and Neurobasal medium (1:1) containing 0.5% N2, 1% B27, 1% GlutaMax, 1% NEAA, 50 µM β-ME, 1% P/S, 20 ng/mL EGF and 20 ng/mL bFGF.
4. Glia Medium: DMEM, 10% FBS.
5. NPC Freeze medium: NPC Medium, 30% KOSR, 10% DMSO, 5 µM Y-27632.
6. hPSC Freeze medium: 90% KOSR, 10% DMSO, 10 µM Y-27632.
7. NDM Medium: Neurobasal medium containing 1% N2, 2% B27, 1% GlutaMax, 1% NEAA, 50 µM β-ME, 10 ng/mL BDNF and 10 ng/mL GDNF.
8. C2 medium: DMEM/F12 and Neurobasal medium (2:1) containing 0.8% N2, 0.4% B27, 0.4 µg/mL L-AA, 5 µM forskolin, 10 ng/mL BDNF and 10 ng/mL GDNF.
9. Blocking buffer: 3% BSA, 2% Triton X-100 in DPBS.
10. PBST: DPBS with 0.1 % Tween-20.
11. Tyrode solution: 150 mM NaCl, 4 mM KCl, 2 mM MgCl_2_, 3 mM CaCl_2_, 10 mM glucose and 10 mM HEPES at pH 7.4 (adjusted with KOH) and 300 mOsm.
12. Intracellular solution: 0.2 mM EGTA, 130 mM K-Gluconate, 6 mM KCl, 3 mM NaCl, 10 mM HEPES, 4 mM ATP-Mg, 0.4 mM GTP-Na, 14 mM phosphocreatine-di(Tris) at pH 7.2 and 285 mOsm.

### 2.9. Differentiation Procedures

hPSCs were routinely cultured with hPSC medium (Nuwacell-ncTarget) and passaged upon ~80% confluency at a 1:10 ratio using dissociation buffer (0.5 mM EDTA).

Notes: It is important that the hPSCs were routinely tested and maintained free of mycoplasma and other contaminations.

#### 2.9.1. NPC Generation from hPSCs

Day 0: Pre-treatment

The protocol was started under a feeder-free culture system. Coat culture dishes or plates with Matrigel (1:200) for at least 2 h at 37 °C in a humidified, 5% CO_2_ incubator. hPSCs were later passaged at a 1:6 ratio by 0.5 mM EDTA, and sub-cultured on Matrigel-coated plates with hPSC medium supplemented with 5 μM Y-27632, one of the ROCK inhibitors. The next day, hPSCs were cultured with fresh hPSC medium without Y-27632, and allowed to grow till reaching the desired confluency.

Notes: Before the neural induction, remove the differentiated colonies as much as possible.

RA vs. SMADi

**Figure 1:**
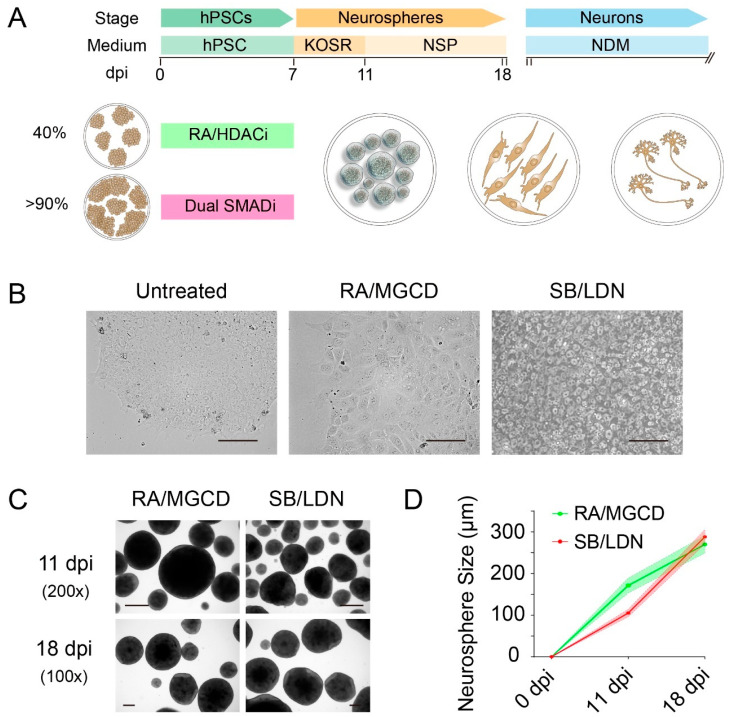
Differentiation strategy for NPC generation and neurosphere formation. (**A**) Differentiation strategy by RA/MGCD and SB/LDN methods. (**B**) Morphology change of hPSCs upon treatment. Scale bar: 100 μm. (**C**,**D**) Neurosphere formation. Scale bar: 100 μm. RA: retinoic acid; MGCD: MGCD0103; SB: SB431542; LDN: LDN193189.

The starting cell confluency is different between the RA and SMADi methods. Basically, ~40% confluency works well with the RA-induced method, whereas >90% confluency is required for the dual SMADi method (Figure 1A). For the RA method, we generally added MGCD0103, one of the histone deacetylase inhibitors (HDACi), together with RA for treatment, since we and others have previously shown that suppression of HDAC, especially HDAC3, can promote neural differentiation of hPSCs [2,3]. For the dual SMADi method, we used SB431542, the TGF-β/SMAD inhibitor, and LDN193189, the BMP/SMAD inhibitor, for treatment. Note that LDN193189 is a derivative of dorsmorphin, and has been used instead of noggin [23,24,25], since it is more economic.

Day 1–Day 7: Neural Commitment

The day when hPSCs were treated with instructive factors was recorded as day 1. Since day 1, replace medium daily with hPSC medium supplemented with: (a) 10 μM RA/0.8 μM MGCD0103; or (b) 10 μM SB431542/0.1 μM LDN193189, respectively. The treatments both lasted for 7 days for fully neural commitment.

Notes: Five days of dual-SMADi treatment might be already sufficient to impose neural fate based on the previous study [23]. In this protocol, 7-day treatment was intended to treat adherent hPSCs thoroughly due to their high-confluency culture, aiming to obtain higher purity of neurospheres.

Day 8: Neurosphere Formation

Aspirate treatment medium and wash hPSCs with DPBS. Add Accutase (e.g., 1 mL for each well of 6-WP) to digest hPSCs for 3–5 min, until cells fall off the plates. Stop the digestion by adding the same volume of DMEM/F12 medium. Collect digested cells into 15 mL conical tubes, and gently pipette up and down 1–2 times. Spin down cells at 800 rpm for 4 min, and discard supernatant. Resuspend hPSCs with KOSR medium supplemented with 10 μM Y-27632. Transfer cells into low-attachment petri dishes for suspension culture.

Notes: For neurosphere formation, hPSCs can also be digested by 0.5 mM EDTA (as clump passage).

Day 10: Medium Change

Collect neurospheres into 15 mL conical tubes. Stand still until all neurospheres sink to the bottom. Discard supernatant and resuspend with KOSR medium without Y-27632. Transfer neurospheres into petri dishes for continual suspension culture.

Notes: Before collecting neurospheres, shake petri dishes in a clockwise direction, until most of the neurospheres gather in the center. Transfer neurospheres very carefully into 15 mL conical tubes, since the spheres might strongly adhere to the inner wall of plastic tips. Using 1 mL wide bore pipette tips should be best.

Day 11: Observation

Observe neurospheres under microscopy, and collect part of spheres for RNA extraction.

Notes: Typical neurospheres were with a central dark core.

Day 12–Day 17: Medium Change

Since day 11, replace KOSR medium with NSP medium every 3 days for further suspension culture, according to the method described at day 10.

Day 18: NPC Culture

Collect neurospheres in 15 mL conical tubes. Discard medium and wash with DPBS once. Add 5 mL pre-warmed Accutase for neurosphere digestion in a 37 °C water bath for 3–5 min, whistling pipetting up and down gently several times to facilitate breaking large clumps. Stop the digestion with the same volume of DMEM/F12 medium. Spin down cells at 800 rpm for 4 min and discard supernatant. Resuspend cells with NPC medium supplemented with 10 μM Y-27632, and culture NPCs on Matrigel-coated culture dishes.

Notes: Should coat culture dishes with Matrigel in advance.

Day 19: NPC Expansion

NPCs were routinely cultured with NPC medium on Matrigel-coated dishes and passaged with Accutase for 2 min at a ratio of 3–6. Parts of NPCs were spun down and collected with TransZol for total mRNA isolation and Q-PCR analysis. Parts of NPCs were seeded on Matrigel-coated coverslips for immunostaining analysis. Till confluency, NPCs can be frozen with NPC Freeze medium, or further differentiated into neurons.

#### 2.9.2. Neural Differentiation from NPCs

Day 1: NPC Seeding

Coat culture plates/coverslips with PLO/LAM: poly-L-ornithine (PLO, 15 μg/mL)/Laminin (1 μg/mL), or with MLF555: Matrigel (1:500)/Laminin (5 μg/mL)/Fibronectin (5 μg/mL). NPCs were digested with Accutase for 2 min, and seeded at a density of 1 × 10^4^ cells/cm^2^ on the coated plates cultured with NPC medium.

Notes: The coating procedure of PLO/LAM takes several days: In a sterile hood, place one sterilized coverslip in each well of a 24-WP. Add 15 μg/mL PLO onto each coverslip. Incubate the plates at 37 °C overnight. The next day, aspirate the PLO and let the coverslips dry at room temperature for 30 min. Wash three times using 1 mL of sterile H_2_O for each well. Leave the plate open in the hood until completely dry. Coat with 1 μg/mL Laminin overnight at 37 °C. Aspirate Laminin and let the plates completely dry. Cover the plates, wrap in foil, label with date and store at −20 °C for up to 4 weeks.

Day 2a: Spontaneous Differentiation

For spontaneous differentiation, replace with NPC medium without bFGF and EGF the next day. Medium was half-replaced every 3 days. Neurons and astrocytes were then induced in the following days, which can be fixed for immunostaining at desired timepoints.

Day 2b: Directed Neural Differentiation

For directed neural differentiation with high efficiency, culture NPCs with NDM medium the next day. Later on, the differentiation medium was routinely half-replaced every 3 days.

Day 2c: MN Differentiation

For MN differentiation with high efficiency, NPCs were transduced with lentiviruses with different MOI, adding 6 μg/mL polybrene. The next day, replace with NDM, or C2 medium or C2 medium containing RA (1 μM) and Pur (1 μM). Later on, the differentiation medium was routinely half-replaced every 3 days.

Notes: The produced lentiviruses will need to be tiered before use. Basically, the titer can reach 1 × 10^8^ TU/mL.

Day 4: Antibiotic selection

For MN differentiation by overexpressing transcription factors, add puromycin (0.2 μg/mL) for selection of transduced cells.

Notes: This makes sure that GFP^+^ (NIL-GFP vector) cells have been simultaneously transduced with the other virus (ISL1-LHX3-Puro vector).

Day 5–Day 6: AraC Treatment

Note that treatment with 5 μM AraC for 24 h at around 5–6 dpi can yield higher purity of neurons.

Day 6: Neuron Replate

For a long-term culture, neurons were passaged by Accutase digestion for 2 min, which was stopped by adding the same volume of DMEM/F12 medium. Spin down cells at 800 rpm for 4 min, and discard supernatant. Resuspend cells with the differentiation medium, and seed onto coverslips coated with a glia layer, which contributes to better neuronal support and electrophysiology. The differentiation medium was routinely half-replaced every 3 days.

Notes: For co-culturing with glia, it is best to prepare primary astrocyte around 7 days before NPC differentiation. Basically, primary astrocytes are digested by 0.25% Trypsin and seeded as 5 × 10^4^ cells on each coverslip until confluency, followed by treatment with 5 μM AraC for 24 h. The treated glial layer is continually cultured with fresh glia medium, which is ready for neuron replate.

Day 12: Can add muscle cells for co-culture

In the case of performing functional experiments of MNs, this timepoint is good for adding differentiated primary muscle cells or C2C12 cells.

Notes: FSK should be removed from C2 medium if muscle cells are added in the culture, since FSK could inhibit the muscle growth. The muscle cells can be observed twitching with MN co-culture (Supplementary Video S1).

Day 21 and later: Analysis

Cultured neurons were fixed for immunostaining or used for electrophysiology detection at desired timepoints.

### 2.10. Immunostaining Analysis

1. Fix NPCs or neurons on coverslips with 4% PFA for 15 min at room temperature.
2. Wash with DPBS 3 times (5 min each time).
3. Incubate with blocking buffer for 30 min at room temperature.
4. Incubate with primary antibodies (diluted with blocking buffer) overnight at 4 °C.
5. Wash with PBST 3 times (5 min each time).
6. Incubate with second antibodies for 1 h at room temperature.
7. Wash with PBST 3 times (5 min each time).
8. Incubate nucleus with Hoechst 33342 for 5–10 min at room temperature.
9. Seal coverslips inverted onto microscope slides with 10% PVA.
10. Observe under the fluorescence microscope.

### 2.11. Q-PCR Analysis

1. Total mRNAs were extracted from NPCs using TransZol reagents according to the manufacturer’s instructions.
2. Total mRNAs were reverse-transcripted into cDNAs using RevertAid First Strand cDNA Synthesis Kit.
3. PCR was performed using PerfectStart Green qPCR SuperMix and carried out on a 96-well ABI QuantStudio Dx.
4. The PCR program consisted of 30 s at 94 °C, followed by 42 cycles of 5 s at 94 °C, 15 s at 62 °C, and 15 s at 72 °C.
5. All primers were verified by melting curve analysis containing a single melt curve peak.
6. Relative gene expression levels were analyzed using the 2^−∆∆Ct^ algorithm normalized to the housekeeping HPRT and relative to control samples.

### 2.12. Electrophysiology

Differentiated neurons on the glia layer were cultured for 35 days and used for electrophysiology detection. Generally, neurons were maintained at 30 °C in a submersion chamber with Tyrode solution, and whole-cell recordings were performed using recording glass pipettes filled with an intracellular solution. Series and input resistance (ranging from 50 to 1500 MΩ) were measured in voltage-clamp with a 400 ms, 10 mV step from a −60 mV holding potential. Action potentials were recorded in current-clamp and elicited by current injections ranging from −20 to 200 pA at 20 pA increments and 400 ms in duration. Currents were filtered at 3 kHz, acquired and digitized at 10 kHz using Clampex10.3 software.

## 3. Results and Discussion

### 3.1. Rapid and Highly Efficient Generation of NPCs from hPSCs

Previously, we performed neural differentiation by using floating EBs and neural rosettes. Around 50% of the area of the adhered cells was filled with neural rosettes positive for PAX6 (Supplementary Figure S1). The other flat cells were neural crest cells, which emerge alongside the edges of colonies (Supplementary Figure S1). To obtain a pure population of NPCs, rosettes need to be manually isolated using scalpels. This method is inefficient and labor-intensive, and can be difficult to reproduce and further causes overall variability.

We then used two sets of instructive factors including RA and dual-SMADi (SB/LDN) to initiate neural induction, respectively (Figure 1A). Seven days treatment directed hPSCs towards the neural lineage, which were further digested and cultured as neurospheres. The morphology of hPSCs changed starting from the outer side of colonies upon treatment, which is more obvious in RA-treated cells with larger nuclei and expanded cytoplasm (Figure 1B).

hPSCs were later digested and cultured as neurospheres in suspension, which facilitates the exposure to other inducing factors such as N2 supplement. It appears that RA induced an earlier neurosphere formation at 11 dpi, whereas SB/LDN induced a more rapid differentiation (Figure 1C,D). During RA treatment, the addition of HDACi such as MGCD0103 can greatly promote the generation of NPCs by 15-fold, as we have previously shown that HDACs, especially HDAC3, are critical regulators in neural differentiation [2]. It is not wise to compare the productivity between those two methods, due to different numbers of starting cells; however, we indeed can harvest more NPCs from one-time differentiation with the SB/LDN method.

**Figure 2:**
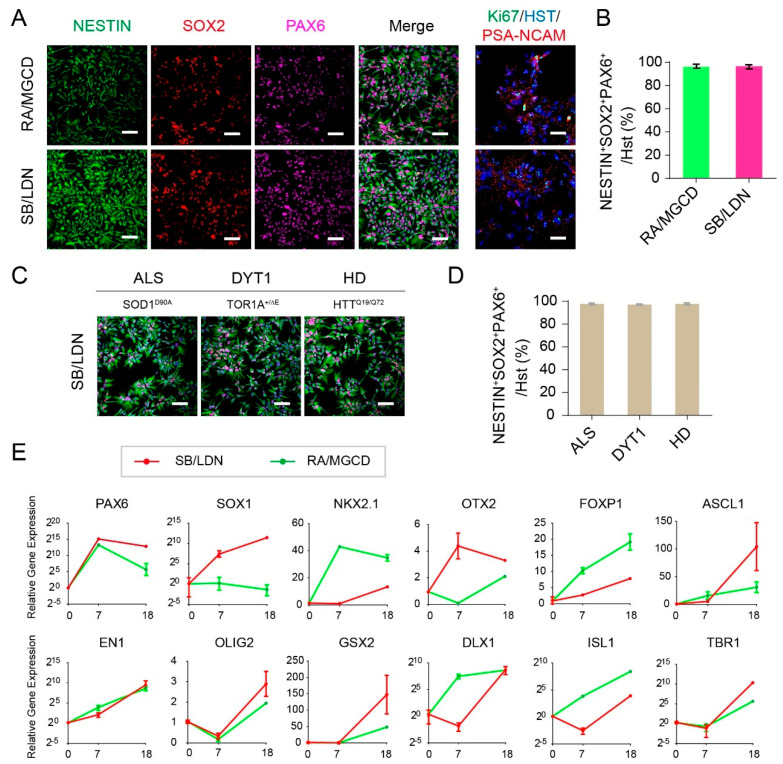
NPC generation and identity characterization. (**A**,**C**) Immunostaining of generated NPCs derived from both healthy individuals and patients with neurodegenerative diseases. Scale bar: 100 μm. (**B**,**D**) Statistics of NPCs with NESTIN/SOX2/PAX6 triple positive. (**E**) Identity characterization of NPCs by Q-PCR.

At 18 dpi, neurospheres were dissociated and cultured as NPCs on the monolayer, which are ready to be frozen for further use. Both methods can give rise to high purity of NPCs, as shown by PAX6/SOX2/NESTIN triple positive (more than 97%), as well as Ki67 and PSA-NCAM positive (Figure 2A,B). Similarly, the hPSCs derived from patients with neurodegenerative diseases, such as amyotrophic lateral sclerosis (ALS; SOD^G90A^ mutation) [30], isolated dystonia (DYT1; TOR1A^∆E^) [10,32] and Huntington’s disease (HD; HTT^Q19/Q72^) [33], can also be induced into NPCs with comparable high efficiencies (Figure 2C,D).

### 3.2. Identity Comparisons of Generated NPCs

We further examined the identities of generated NPCs by Q-PCR (Figure 2E). Both methods can dramatically induce PAX6 expression, reminiscent of human telencephalon identity. Despite their induction capacity, there are indeed differences between the two methods. The SB/LDN method is in general superior to the RA/MGCD method, in terms of inducing higher progenitor markers such as PAX6, SOX1, OTX2, ASCL1, OLIG2, GSX2 and TBR1 (Figure 2E). However, NPCs induced from the RA/MGCD method possess a unique identity, which is expressed as higher NKX2.1, FoxP1 and ISL1 (Figure 2E). Since NKX2.1 is a transcriptional marker for medial ganglionic eminence (MGE), the main site of GABAergic neurogenesis, the RA/MGCD method induced NPCs with more ventral-like characters.

Notably, SOX1 expression can hardly be induced by RA/MGCD treatment (Figure 2E), which greatly compromised the full capacity of neural induction by the RA signaling. This might be due to the high concentration of RA that we used, since varying concentration of RA may be responsible for generating NPCs with different potential [34]. RA has been shown to play important roles in neural development, especially in patterning of the neural plate with a gradient of RA in the antero-posterior axis [21]. RA also participates in multiple aspects of neural activities, such as axon regeneration and neural differentiation. Initially, RA was commonly used for neural induction in mouse ESCs, and was later adopted into the hPSC neural differentiation with varying concentrations between 1 and 10 μM in the medium by different research groups [13,35,36,37,38]. Interestingly, one study has also combined RA with SMADi to promote the directed differentiation of hESCs to cerebral cortex stem and progenitor cells [22]. Without RA, however, cortical neural induction from hPSCs by dual SMADi was inefficient [22]. The NPC cultures derived by this method expressed high levels of FOXG1 and EMX1, and subsequently TBR2, but did not express any ventrally or caudally expressed markers such as DLX1, NKX2.1, HOXB4 and ISL1 [22]. Therefore, RA induction is more likely to bring about heterogeneity of neural differentiation, based on different timepoints and dosages for treatment.

### 3.3. Spontaneous Neural Differentiation of Generated NPCs

**Figure 3:**
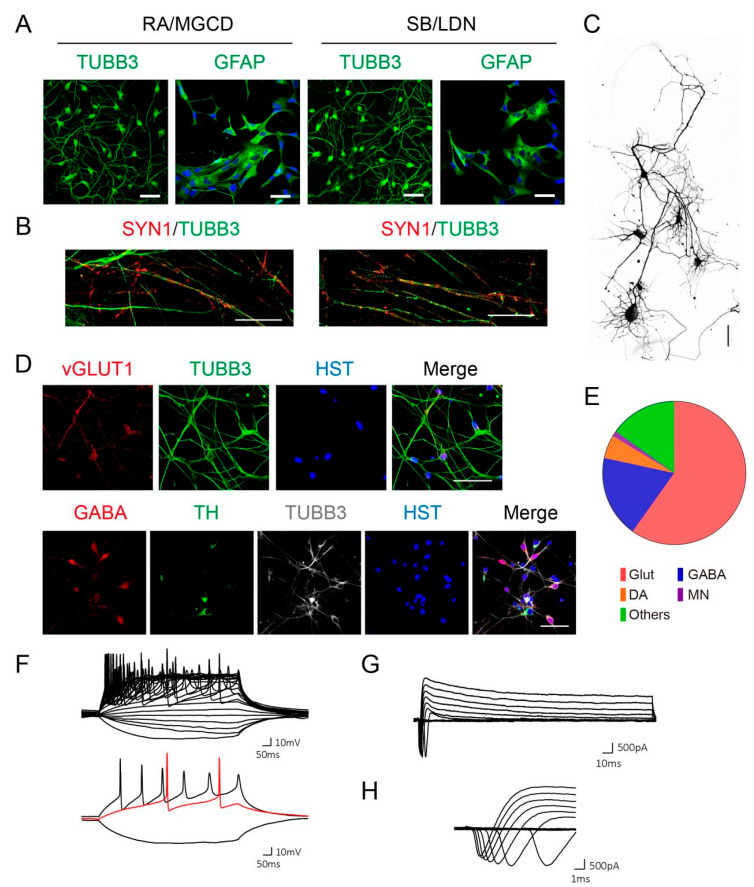
Spontaneous neural differentiation from generated NPCs. (**A**) NPCs were spontaneously differentiated into neurons and astrocytes, represented by TUBB3 and GFAP staining, respectively. Scale bar, 50 μm. (**B**) Synapse formation by SYN1 staining of differentiated neurons at 30 dpi. Scale bar: 25 μm. (**C**) Complicated morphology of differentiated neurons at 30 dpi. Scale bar: 20 μm. (**D**,**E**) Neural subtypes differentiated from SB/LDN NPCs. Scale bar: 50 μm. (**F**) Repetitive action potential waveforms recorded by whole-cell patch-clamp recording. (**G**) Representative waveform of inward and outward currents. (**H**) A zoomed-in view of inward currents, resembling sodium currents. (**F**–**H**) Adapted from our previous results [9].

Nevertheless, NPCs from both methods can be spontaneously induced into neurons and astrocytes with comparable efficiencies (Figure 3A). The differentiated neurons can be further cultured on a glial layer and can become very mature with complicated morphology and axonal connections (Figure 3B,C). Since the generated NPCs by the current methods are basically with forebrain identities, they were differentiated into the majority of excitatory (GLUT^+^) and inhibitory (GABA^+^) neurons, at a similar ratio of excitatory/inhibitory neurons inside the brain, but hardly motor neurons (MNs) or dopaminergic neurons (DAs) (Figure 3D,E). Whole-cell patch-clamp recordings showed that differentiated neurons fired repetitive action potentials (APs) upon current injection (Figure 3F), and displayed inward and outward currents, reminiscent of sodium and potassium currents through voltage-gated sodium and potassium channels, respectively (Figure 3G,H).

As an expanded advantage, the SB/LDN method can work synergistically with other inhibitors or instructive factors targeting SHH pathway or WNT pathway, such as Pur, SHH, CHIR99021 and FGF8 [18,39,40,41,42,43,44,45], for further rostro-caudal patterning and dorso-ventral patterning. The generated NPCs would be endowed with regional identities that can further be induced as multiple neural subtypes with varying efficiencies, depending on which subtype. For instance, the efficiency for MN subtype would be lower. It is notable that the optimal concentration of each factor will need to be tested by examining its dose-dependent responses for inducing neuron subtypes of interest. As a roundup, the comparison of strategies for generating NPCs from hPSCs is listed in Table 1.

### 3.4. Rapid and Highly Efficient Generation of MNs from NPCs

**Figure 4:**
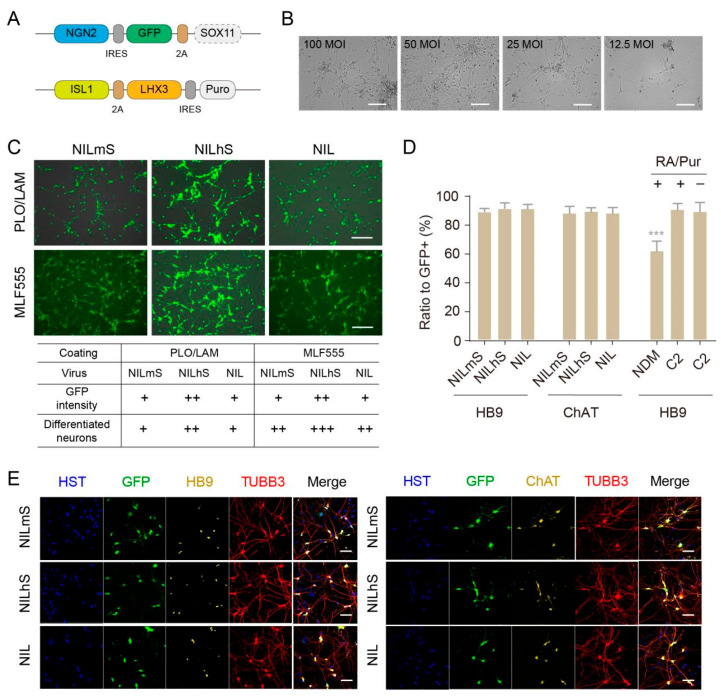
MN differentiation from generated NPCs. (**A**) Sets of transcription factors for MN differentiation. (**B**) The MOI of virus usage. Scale bar: 100 μm. (**C**) Neural differentiation with different sets of transcription factors and coating conditions. +, ++, +++ represent the scales of neuron number or GFP intensity. Scale bar: 100 μm. (**D**) Efficiencies of MN differentiation. *** *p* < 0.001. (**E**) Immunostaining of MN markers. Scale bar: 50 μm. Pur: purmorphamine; MLF555: Matrigel (1:500)/Laminin (5 μg/mL)/Fibronectin (5 μg/mL); PLO/LAM: PLO (15 μg/mL)/Laminin (1 μg/mL); NIL: *NGN2*, *ISL1* and *LHX3;* NILmS: *NGN2*, *ISL1*, *LHX3*, *mSOX11*; NILhs: *NGN2*, *ISL1*, *LHX3*, *hSOX11*; C2: C2 medium; NDM: NDM medium.

Among all reported studies for subtype differentiation, MN induction has long been tricky. Many endeavors have been devoted to developing MN differentiation, such as the standard step-by-step protocols by EBs/MN patterning [27,28]; however, the overall efficiencies are still low. The neural differentiation by introducing transcription factors, such as NGN2, directly into hPSCs has provided an alternative avenue to rapidly differentiating neurons [29], but with the caveat that it is still a one-time process without any intermediate stage for cell stock. We thus, based on our established preservable NPC platform, further differentiated MNs by introducing transcription factors that govern the development of MNs, such as NGN2, ISL1 and LHX3, according to our and others’ previous studies [9,31]. Moreover, another transcription factor, SOX11, functions as survival factors during spinal cord development [46,47] and is used to convert adult human fibroblasts into cholinergic neurons [48]. Therefore, we refined sets of transcription factors (*NGN2*, *ISL1* and *LHX3*; or say “NIL”), with or without the addition of *SOX11*, packaged into two lentiviral plasmids, respectively (Figure 4A). We also distinguished the usage of human *SOX11* (hSOX11) and mouse *SOX11* (mSOX11), since they possess differential effects on direct reprogramming such that mSOX11, but not hSOX11, successfully reprogrammed primary human fibroblasts into MNs [48]. Meanwhile, we also improved several culture conditions specifically for this transcription-factor-mediated MN differentiation.

We thus transduced three groups of lentivirus (NIL, NILhS, NILmS), respectively, in NPCs. First, we tested the amounts of virus usage. The optimal multiplicity of infection (MOI) would be 50 for each virus, which renders the best survival of differentiated neurons (Figure 4B). Second, we examined the coating conditions. The results showed that the Matrigel/LAM/Fib (MLF555) coating outperformed the classic PLO/LAM coating in supporting both neural differentiation and survival (Figure 4C). Notably, the NILhS group gave rise to higher GFP signals and, more importantly, many more surviving neurons, indicating the superior effect of hSOX11, rather than mSOX11 or without SOX11, in promoting neural differentiation (Figure 4C). Interestingly, by 21 dpi, the surviving neurons from all groups expressed comparable high ratios of MN markers including HB9^+^ /GFP^+^ (88.2% ± 3.5%) and CHAT^+^/GFP^+^ (86.5% ± 4.1%), suggesting that SOX11 promoted MN survival but did not affect the specification of MN subtype (Figure 4D,E).

Third, we also tested different culture mediums for MN differentiation. We found that C2 medium induced significantly higher ratios of MNs, compared to the ratio of HB9^+^/GFP^+^ (61.4% ± 8.2%) with NDM medium that was used for pan-neural differentiation (Figure 4D). Moreover, adding RA/Pur, the MN patterning factors, into C2 medium did not produce significant advantages (Figure 4D), suggesting that the caudalization and ventralization patterning might not be required under the condition of transcription-factor-induced MN differentiation. This actually renders economic advantages for researchers. Besides, to circumvent the persistent expression of transcription factors, we also made use of inducible expression (NIL in one vector under the tetO promoter) by doxycycline treatment, which performed similar MN differentiation as indicated by the infection of pLenti-Hb9-GFP [49] (Supplementary Figure S2). Taken together, we refined the MN differentiation from NPCs with ~90% efficiencies by introducing transcription factors, especially hSOX11, whilst improving the culture conditions.

### 3.5. Modeling MNDs by Differentiated MNs

**Figure 5:**
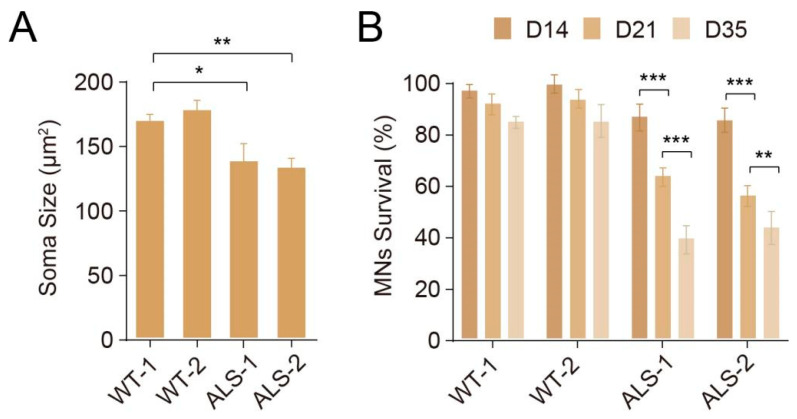
Modeling ALS using differentiated MNs. WT-and ALS-NPCs were differentiated into MNs, respectively. Under the stress by withdrawal of neurotrophic factors: (**A**) Soma size (μm^2^) of WT- and ALS-NPCs-derived MNs. * *p* < 0.05; ** *p* < 0.01. (**B**) Survival of WT- and ALS-NPCs-derived MNs. ** *p* < 0.01; *** *p* < 0.001.

We further used differentiated MNs for modeling MNDs, such as ALS. Specifically, we obtained MNs from two wildtype (WT) and two ALS (harboring SOD1 D90A mutation) hPSC lines, by NILhS induction. By withdrawal of neurotrophic factors such as BDNF and GDNF, ALS-MNs displayed significant reduction in soma size (Figure 5A), and were more vulnerable to this stress condition, even with the survival effect of hSOX11 overexpression, resulting in gradual and extensive neuronal death (Figure 5B). Interestingly, this strategy for MN differentiation could be further modified to obtain disease-relevant cells. For example, De Santis et al. converted human iPSCs into cranial MNs, which were primarily affected by a severe form of ALS with bulbar onset and worst prognosis [50]. Specifically, they overexpressed NGN2, ISL1 and PHOX2A in iPSCs and eventually obtained electrophysiological active cranial MNs, at comparable efficiencies (>90%) with our method for spinal MNs. While they started the differentiation from hPSCs, it is highly possible that preservable NPCs in our protocol can also be induced into multiple subtypes of MNs, by replacing LHX3 with other subtype factors, to precisely model the disease pathogenesis.

Overall, the results suggest that differentiated MNs by the induction of transcription factors can be used to model the pathology of MNDs at multiple aspects, if not all, and might provide an excellent cell platform for further high-throughput studies and drug screening.

## Figures and Tables

**Table 1:** Comparison of strategies for generating NPCs from hPSCs.

Methods	Instructive Factors	Starting Density	Treatment Days	Purity (Pax6^+^/Sox2^+^/Nestin^+^)	Induction Capabilities	Differentiation Capabilities	Notes
Dual SMAD inhibition	SB431542 (10 μM) LDN193189 (0.1 μM)	>90% hPSCs	7 days	>97%	Superior in general	Both neurons and astrocytes	Work with other patterning factors (such as purmorphamine, CHIR99021, FGF8, etc.) to generate region-specific NPCs
RA treatment	RA (10 μM) MGCD0103 (0.8 μM)	~40% hPSCs	7 days	>97%	Superior in inducing NKX2.1, but incapable of inducing SOX1 with 10 μM RA	Both neurons and astrocytes	MGCD0103 can be replaced as other HDACi such as sodium butyrate, trichostatin A and valproic acid

## Data Availability

Data available on request from the corresponding author.

## Supplementary Materials

The following are available online at https://www.mdpi.com/article/10.3390/cells10113087/s1, Figure S1. Formation of neural rosettes; Figure S2. MN differentiation by inducible transcription factors; Video S1. Co-culture of MNs and differentiated C2C12 muscle cells.

## Appendix A

**Table A1:** Antibodies used for immunostaining.

Primary Antibody	Species	Dilution	Source
NESTIN	Ms	1:400	Millipore, #MAB5326
PAX6	Rb	1:500	Sigma, #HPA030775
SOX2	Gt	1:50	Santa Cruz, #sc-17320
Ki67	Rb	1:1000	Leica, #NCL-Ki67p
PSA-NCAM	Ms	1:250	DSHB, #5A5-a
TUBB3	Rb	1:500	ABclonal, #A17074
TUBB3	Ms	1:750	Biolegend, #801201
GFAP	Rb	1:200	ABclonal, #A14673
SYN1	Rb	1:200	Cell Signaling, #5297
vGLUT1	Ms	1:500	Synaptic System, #135303
GABA	Rb	1:1000	Sigma, #A2052
TH	Ck	1:1000	Aves, #TYH
HB9	Ms	1:100	DSHB, 81.5C10-c
ChAT	Gt	1:200	Millipore, #AB144P
GFP	Ck	1:1000	Aves, #GFP-1020
Secondary Antibody			
Donkey anti-Mouse IgG (H + L), Alexa Fluor 488		1:500	Invitrogen, #A-21202
Donkey anti-Rabbit IgG (H + L), Alexa Fluor 488		1:500	Invitrogen, #A-21206
Goat anti-Chicken IgY (H + L), Alexa Fluor 488		1:500	Invitrogen, #A-11039
Donkey anti-Mouse IgG (H + L), Alexa Fluor 555		1:500	Invitrogen, #A-31570
Donkey anti-Rabbit IgG (H + L), Alexa Fluor 555		1:500	Invitrogen, #A-31572
Donkey anti-Goat IgG (H + L), Alexa Fluor 555		1:500	Invitrogen, #A-21432
Goat anti-Mouse IgM (H), Alexa Fluor 555		1:500	Invitrogen, #A-21426
Donkey anti-Mouse IgG(H + L), Alexa Flour 647		1:500	Invitrogen, #A-31571
Donkey anti-Rabbit IgG(H + L), Alexa Flour 647		1:500	Invitrogen, #A-31573
Donkey anti-Goat IgG (H + L), Alexa Fluor 647		1:500	Invitrogen, #A-21447

## Appendix B

**Table A2:** Primer sequences for Q-PCR.

Gene	Forward Primer (5′–3′)	Reverse Primer (5′–3′)	bp
*HPRT*	GCTTTCCTTGGTCAGGCAGTA	GTCTGGCTTATATCCAACACTTCGT	94
*ASCL1*	GTCCTGTCGCCCACCATCTC	CCCTCCCAACGCCACTGAC	251
*DLX1*	TGCCAGAAAGTCTCAACAGCC	CGAGTGTAAACAGTGCATGGA	125
*EN1*	CGTGGCTTACTCCCCATTTA	TCTCGCTGTCTCTCCCTCTC	117
*FOXP1*	CTACCGCTTCCATGGGAAATC	CTGTTGTCACTAAGGACAGGG	207
*GSX2*	ATGTCGCGCTCCTTCTATGTC	CAAGCGGGATGAAGAAATCCG	106
*ISL1*	GCGGAGTGTAATCAGTATTTGGA	GCATTTGATCCCGTACAACCT	102
*NKX2.1*	AGCACACGACTCCGTTCTC	GCCCACTTTCTTGTAGCTTTCC	68
*OLIG2*	AGCTCCTCAAATCGCATCC	AAAAGGTCATCGGGCTCTG	146
*OTX2*	CAAAGTGAGACCTGCCAAAAAGA	TGGACAAGGGATCTGACAGTG	179
*PAX6*	GCCCTCACAAACACCTACAG	TCATAACTCCGCCCATTCAC	149
*SOX1*	GCGGAGCTCGTCGCATT	GCGGTAACAACTACAAAAAACTTGTAA	62
*TBR1*	GCAGCAGCTACCCACATTCA	AGGTTGTCAGTGGTCGAGATA	76
