# Supplementary material for: A Step-by-Step Refined Strategy for Highly Efficient Generation of Neural Progenitors and Motor Neurons from Human Pluripotent Stem Cells
Source: Cells. 2021 Nov 9;10(11):3087. doi: 10.3390/cells10113087 (PMC8625124; doi:10.3390/cells10113087)
Supplement: Supplementary file 1 [file cells-10-03087-s001.zip › cells-1411770-supplementary.pdf]

Supplementary Figures

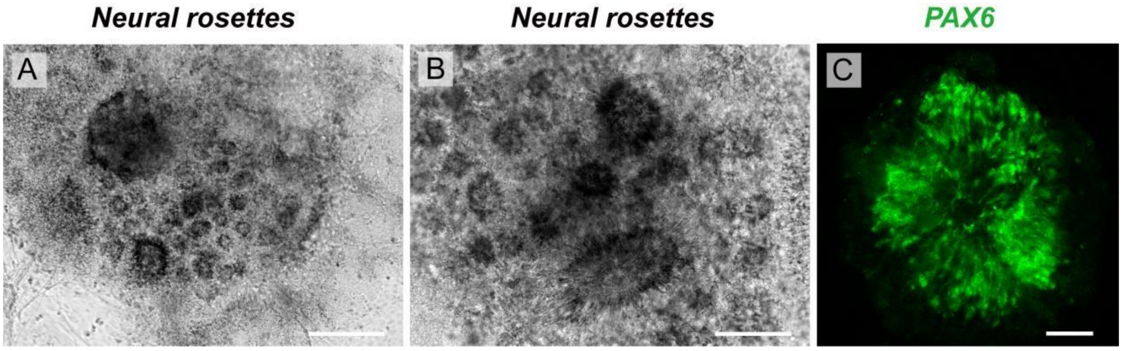

Figure S1. Formation of neural rosettes.
(**A**) Neural rosettes (Scale bar: 500 μm) and (**B**) enlarged (Scale bar: 200 μm). (**C**) PAX6 staining for neural rosettes (Scale bar: 50 μm).

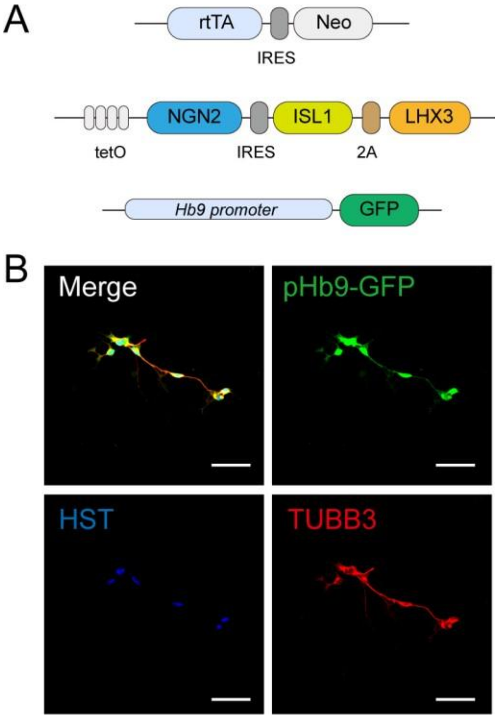

Figure S2. MN differentiation by inducible transcription factors.
(A) Inducible sets of transcription factors for MN differentiation. (**B**) MN differentiation indicated by the infection of pLenti-Hb9-GFP. Scale bar: 50 μm.
